# Treatment of *Saccharomyces cerevisiae* with cigarette smoke extract causes vacuolar fragmentation to combat cigarette smoke-induced cellular toxicity

**DOI:** 10.3389/ftox.2026.1835170

**Published:** 2026-07-22

**Authors:** Aditya Shukla, Srimonti Sarkar, Alok Kumar Sil

**Affiliations:** 1 Department of Microbiology, University of Calcutta, Kolkata, India; 2 Department of Biological Sciences, Bose Institute, Kolkata, India

**Keywords:** cigarette smoke, mitochondria, phospholipase, PI (3,5) P2, vacuolar fragmentation

## Abstract

Exposure to cigarette smoke is one of the major risk factors for developing various diseases, such as chronic obstructive pulmonary disease (COPD), cardiovascular disorders, and cancer mediated via cellular oxidative stress and organelle dysfunction. To this end, the current study investigated how cigarette smoke extract (CSE) affects the vacuole structure and function in *Saccharomyces cerevisiae* as the vacuole plays a crucial role in handling oxidative stress-induced misfolded proteins. Our results indicated that CSE exposure causes transient vacuolar fragmentation peaking at 0.5 h, which correlates with reduced protein misfolding and improved cell survival. However, excessive fragmentation or vacuolar fusion sensitizes cells toward CSE-mediated cellular toxicity. To understand the underlying mechanism, this study provides the evidence of the involvement of PI(3,5)P2-Mediated signaling and phospholipase-driven remodeling of lipid moieties and suggests a role for mitochondrial function in CSE-mediated vacuolar fragmentation. Prolonged exposure to CSE impairs mitochondrial function and thus disrupts fragmentation, the adaptive survival strategy against cigarette smoke (CS). It results in proteostasis collapse, a characteristic shared by many inflammatory and degenerative disorders. Together, this study reveals a previously unrecognized cellular protection mechanism induced by CS and suggests avenues for further investigation for mitigating CS-mediated diseases.

## 1 Introduction

Cigarette smoke (CS) is a mixture made up of more than 7,000 different chemicals, such as heavy metals, aldehydes, various reactive oxygen and nitrogen species (ROS and RNS), and several polycyclic aromatic hydrocarbons (Rodgman, and Perfetti, 2008). These oxidants include relatively stable organic radicals, such as semiquinones, and short-lived forms, such as superoxide anions (Magder, 2006). These substances damage proteins, lipids, and nucleic acids, either directly or indirectly causing oxidative stress. In addition to its contents, CS can induce the generation of various oxidants at the cellular level. It reacts chemically with biomolecules to produce more oxidants, activates endogenous ROS-producing systems, and concurrently compromises antioxidant defenses (Seo et al., 2023). Thus, in contrast to single-agent oxidants such as hydrogen peroxide (H_2_O_2_), CS-mediated oxidative stress is more robust and, due to its multidimensional action, overwhelms cellular redox equilibrium. Therefore, exposure to CS is associated with significant cellular damage and an increased risk of conditions such as atherosclerosis, cancer, and chronic obstructive pulmonary disease (COPD) (Timothy and Nneli, 2007; Walser et al., 2008).

Oxidative stress and proteotoxicity induce adaptive remodeling of cellular compartments, including organelles, by altering fundamental homeostatic processes. Many organelles undergo membrane fission and fusion events during cell division, vesicular traffic, or in response to environmental changes. A few examples include the lysosome (Ward et al., 1997), peroxisomes (Kuravi et al., 2006), mitochondria (Bleazard et al., 1999), and the Golgi apparatus (Aufschnaiter and Büttner, 2019). The morphology of vacuoles dynamically adjusts to intra- and extracellular environmental conditions by fission (also known as fragmentation) and fusion, resulting in the formation of either multiple smaller vacuoles or a single larger vacuole, respectively. For instance, in *Saccharomyces cerevisiae*, vacuole fragmentation is caused by lactic acid stress, endoplasmic reticulum stress, or hyperosmotic shock, whereas vacuole fusion is induced by hypotonic shock, nutrient limitation, or TORC1 inactivation (Bonangelino et al., 2002; Acharya et al., 1998). Vacuolar fragmentation also occurs during cell division, so that vacuoles can be partitioned into daughter cells (Weisman, 2003).

In S. *cerevisiae*, vacuoles play roles in osmoregulation, autophagy, and the storage of various ions and amino acids (Klionsky et al., 1990). Lysosomal disorders are caused by mutations in genes that affect lysosomal degradation, indicating the importance of regulating lysosomal/vacuolar hydrolytic capacity to the proper functioning of eukaryotic cells (Lawrence et al., 2010). It has been previously reported that oxidative stress causes the accumulation of misfolded proteins. Under this stressed condition, vacuolar/lysosomal function (autophagy), in tandem with the ubiquitin-proteasome system, is vital to maintain proteostasis (Gao et al., 2019). In this context, a recent report has shown enhanced microautophagic activity upon vacuolar fragmentation; however, fragmentation does not influence the macroautophagic activity (Takuma and Ushimaru, 2022). Therefore, fragmentation, which increases the surface area of vacuolar membranes, might play an important role in clearing accumulated misfolded proteins. This could be another cellular mechanism to eliminate the toxic effects resulting from the accumulation of misfolded proteins. Since cigarette smoke extract (CSE)-mediated oxidative stress promotes protein misfolding, and many CSE-associated diseases are caused by an accumulation of misfolded proteins, the current study investigated the effect of CS-induced oxidative stress on vacuolar structure and function using the yeast *S. cerevisiae* as a model organism. It has been used because it uses pathways that are evolutionarily conserved, such as endocytosis, membrane transport, oxidative stress response, and protein quality control; it is also experimentally tractable (Maiti et al., 2021; Shukla et al., 2025).

Moreover, phosphoinositide signaling mediated by PI(3,5)P2, which regulates vacuolar dynamics in yeast, is evolutionarily conserved and functionally related to lysosomal regulation in mammals. Given the central role of lysosomal dysfunction and proteostasis imbalance in neurodegeneration, cancer, and smoke-related diseases, yeast serves as a valuable model for studying conserved cellular mechanisms underlying these pathologies.

Our results demonstrate that CSE treatment induces early vacuolar fragmentation, which enhances the clearance of misfolded proteins and improves cell survival. However, excessive fragmentation was shown to be detrimental.

## 2 Materials and methods

### 2.1 Preparation of CSE

CSE was prepared using a commercially available filter-tipped cigarette with a tar content of 15 mg and a nicotine content of 1 mg, manufactured by the Indian Tobacco Company Limited (ITC Ltd., India) as previously described (Maiti et al., 2021; Shukla et al., 2025). The smoke from a cigarette was bubbled through 1 mL of 50 mM PBS, pH 7.4, till the cigarette was consumed completely. The pH of this aqueous CSE was adjusted to 7.4 using NaOH and was filter-sterilized by passing the extract through a 0.2 μM syringe filter. The OD 0.6–0.8 was considered 100% and was diluted as per requirements. Moreover, the extract was then lyophilized and weighed. Simultaneously, 1 ml of PBS without CS was also lyophilized and weighed. Approximately, PBS without CS weighed 15.5 ± 0.2 mg, and PBS with CS weighed 16.9 ± 0.3 mg. Therefore, 1 ml of CSE contains ∼1.5 mg of CS components, and this is equivalent to 100%. In all the experiments, freshly prepared 15% CSE (equivalent to ∼0.225 mg/mL) was used.

### 2.2 Yeast strain, media, and CSE treatment

*S. cerevisiae* strain BY4741 (MATahis3Δ1 leu2Δ0 *met15*Δ0 ura3Δ0) was used in this study. All the null mutants used in this study have a BY4741 genetic background and were obtained from Prof. Maya Schuldiner (Weizmann Institute of Science, Israel). HSP104-GFP also has a BY4741 genetic background, and it was a kind of gift from Prof. Markus Tamas (University of Gothenburg, Sweden). Yeast cells were grown in the synthetic dropout medium containing 2% glucose and 0.1% amino acid mixture (containing arginine, 0.9 g; methionine, 0.9 g; tyrosine, 1.35 g; isoleucine, 1.35 g; phenylalanine, 1.35 g; aspartic acid, 4.5 g; glutamic acid, 4.5 g; valine, 4.5 g; threonine, 4.5 g; and serine, 4.5 g) along with 0.005% lysine. For the CSE treatment, based on the previous reports (Maiti et al., 2021; Shukla et al., 2025), the exponentially growing *S. cerevisiae* cells were treated with 15% CSE for different periods.

### 2.3 Cell viability assay

Exponentially growing *S. cerevisiae* cells (10^6^/mL) were treated with 15% CSE for 0.5, 1, and 2 h in the presence or absence of additional reagents as required for the specific experiments. The cells were centrifuged, resuspended in fresh medium, and serially diluted. Thereafter, an aliquot from the serially diluted samples was spotted on the appropriate agar plates. The plates were incubated at 30 °C for 48 h.

### 2.4 Colony-forming unit assay

Exponentially growing *S. cerevisiae* cells (10^6^/mL) were treated with 15% CSE for different periods, with or without additional reagents as required for the specific experiments. The cells were harvested, resuspended, and serially diluted in fresh medium. Thereafter, 100 μL was spread onto agar plates. The plates were then incubated at 30 °C for 48 h. Colonies were counted, and CFU/mL was calculated.

### 2.5 Determination of protein misfolding by ANS dye

The misfolded protein level was spectrophotometrically measured as described by Hawe et al. (2008). Briefly, cells were grown to a midlog phase, and the OD value was set to 0.1. The cells were treated with 15% CSE for 2 h. Then, cell lysates were prepared using lysis buffer (Tris-HCl-50 mM, EDTA-5 mM, NaCl-250 mM, NP-40%–0.1%, DTT-1.5 mM, PMSF-1 mM, protease inhibitor cocktail-1:1000). Furthermore, protein concentration was measured by the Lowry method (Waterborg, 2009). An aliquot containing 30 μg of protein was taken from each lysate to determine protein misfolding using ANS dye. ANS dye was added to each lysate at a final concentration of 30 μM, and the samples were incubated in the dark at room temperature for 30 min. Fluorescence was measured using a spectrofluorometer (Hitachi F7000, Japan) (excitation, 420 nm; emission, 450–550 nm). Background fluorescence was taken into consideration using appropriate unstained and dye-only controls.

### 2.6 FM4-64, CMAC, and rhodamine staining

For FM4-64 staining, cells were stained with FM4-64 before CSE treatment. The cells were grown to a logarithmic phase and centrifuged. The pellet was resuspended in 100 μL of YPD (yeast extract 1%, peptone 2%, dextrose 2%), and 0.5 μL FM4-64 solution (30 µM) was added. It was kept in the dark under shaking conditions for 45 min. The cells were then spun down, washed, and resuspended in 850 μL of YPD medium and kept for 1 h. Thereafter, 150 μL of CSE was added to it to reach a final concentration of 15%, and the mixture was incubated for different periods as indicated in the figures, followed by washing with YCM medium, and visualized using a fluorescence microscope (Olympus IX 71, Japan).

For CMAC staining, exponentially growing cells were treated with CSE as mentioned in the previous section, washed, and then incubated with CMAC at a final concentration of 5 μM at room temperature (30°C) in the dark for 30 min. The cells were then washed and visualized using a confocal laser scanning microscope (Stellaris, Leica Microsystems).

For rhodamine staining, cells were treated with CSE as before, washed, and then incubated with Rhodamine 123 (final concentration 5 mg/mL) for 20 min. The cells were centrifuged, washed, resuspended in PBS, and visualized using a confocal laser scanning microscope (Stellaris, Leica Microsystems).

Background fluorescence was taken into consideration using appropriate unstained and dye-only controls for each experiment.

### 2.7 Fluorescence and confocal microscopy

To visualize GFP-tagged proteins, FM4-64, CMAC, and Rhodamine 123 dye-stained cells, either a regular fluorescence microscope (Olympus IX 81, Japan) or a confocal laser scanning microscope (Stellaris, Leica Microsystems) was used, as described in the Results section. For GFP-tagged proteins and Rhodamine 123 dye-stained cells, the FITC filter was set at an excitation wavelength of 395 nm and an emission wavelength of 510 nm. For FM4-64-stained cells, the TRITC filter was set at 515 nm for excitation and at 640 nm for emission.

### 2.8 Estimation of phosphatidic acid

Phospholipids were extracted from the cell lysate using a methanol-diethyl ether-saturated NaCl mixture (1:1:1, v/v/v). After phase separation, the diethyl ether fraction was collected. An aliquot of it was loaded on an HPLC column. An HPLC machine (Waters, Singapore) consists of a 1525 binary HPLC pump and a 2475 multiwavelength fluorescence detector. The reverse-phase C8 (4.6 mm × 250 mm; Waters) analytical column was used. The mobile phases were a mixture of methanol, acetonitrile, and water (90.5: 2.5,7, v/v/v). A pure phosphatidic acid (PA) solution was used as standard.

### 2.9 Image analysis and quantification

Vacuolar morphology and fragmentation were determined using automated image analysis software ImageJ (NIH). The images were analyzed using consistent thresholding criteria across all experimental groups. For each sample, several random fields were analyzed, and a minimum of 150 cells were counted for each group. The number of vacuoles, their size, and total surface area were calculated using the same ImageJ software. When appropriate, blind analysis was conducted to avoid bias.

### 2.10 Measurement of oxygen consumption rate

For measurement of the whole cell respiration rate, yeast cells were grown in either YCM broth harvested at a late log phase (OD_600_ = 1), followed by resuspension in fresh media at a concentration of 10^8^ cells·mL^−1^. The OCR was measured at 30° C using an Oxytherm fitted with a Clark electrode (Hansatech, United Kingdom). Respiration was measured for 4 min and blocked by adding 1 mM sodium azide (a specific inhibitor of cIV). The average oxygen consumption rate (OCR) (nmol of oxygen produced/min/10^8^ cells) was estimated from the data of at least three biological replicates (n = 9). The baseline and the nonspecific OCR (after sodium azide addition) were negligible.

### 2.11 Statistical analysis

All experiments were conducted in at least three independent biological replicates, unless specified otherwise. Values were calculated as mean ± SD. Statistical analyses were conducted using GraphPad Prism software version (Version 8.0.2 (263)).

If the analysis was based on a single parameter (time series or different treatments, etc.), the test conducted included one-way ANOVA followed by Tukey’s multiple comparisons test if the variances were similar or by the Games–Howell test if the variances were unequal.

For an experiment with two parameters (genotype and time, treatment and time, etc.), two-way ANOVA was performed, followed by an appropriate multiple-comparison test. Differences were considered statistically significant at *p* < 0.05.

## 3 Results

### 3.1 Treatment of *S. cerevisiae* cells with cigarette smoke extract induces vacuolar fragmentation

**FIGURE 1:**
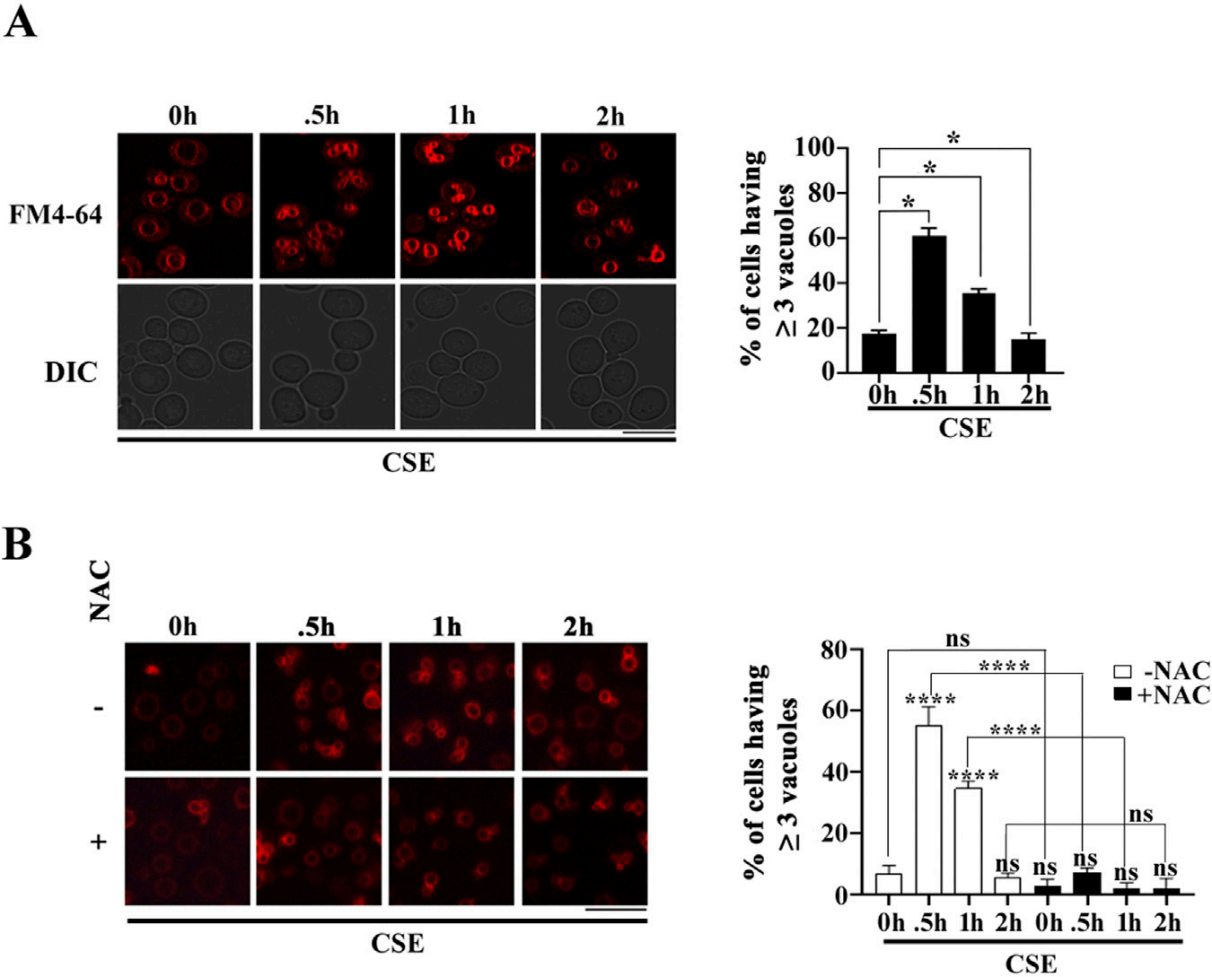
Cigarette smoke extract (CSE)-induced oxidative stress causes vacuolar fragmentation: **(A)** CSE-induced vacuole fragmentation profile. Cells were grown till the log phase and were then stained with FM4-64 for 30 min, followed by chasing for 1 h in YPD media. The cells were then treated with CSE for different time periods as indicated and visualized under a microscope. The quantification of the vacuolar number per cell (n ≥ 150 cells per condition). The bar diagrams on the right represent the quantitative profile. **(B)** N-Acetylcysteine (NAC) prevents CSE-induced vacuolar fragmentation. Cells were grown till the log phase and were then stained with FM4-64 for 30 min, followed by chasing for 1 h in YPD media. The cells were then differentially treated with CSE and 10 mM NAC for different time periods as indicated and visualized under a fluorescence microscope. Each image is representative of 10 different fields. The quantification of the vacuolar number per cell (n ≥ 150 cells per condition). The bar diagrams on the right side represent the quantitative profile. Scale bars: 8 μm. Results are expressed as the mean ± SD of three or more independent experiments. The statistical analyses were conducted via one-way ANOVA. In case of equal variance, standard one-way ANOVA and Tukey’s test were employed **(A)**. Statistical analysis was performed using two-way ANOVA **(B)**, followed by Tukey’s multiple comparisons test. The analysis was performed both within conditions and between −NAC and +NAC conditions at each time point separately. The horizontal bars show comparisons between the two groups at specific times, whereas the asterisks above the bars denote comparisons among different time points within each condition. Significance levels are shown as follows: n.s., not significant; *p < 0.05; **p < 0.01; ***p < 0.001; ****p < 0.0001.

To investigate the effect of CS on vacuolar physiology, yeast cells were treated with 15% CSE for different periods as indicated in the figure, and the vacuoles were stained with FM4-64 dye, which is widely used to stain the vacuolar membrane (Vida and Emr, 1995). The CSE concentration was selected based on the findings from our previous work (Maiti et al., 2021). We also verified it in this study. For this, *S. cerevisiae* cells were treated with various concentrations of CSE (10, 15, and 20%) for different periods (0.5, 1, and 2 h). It was found that growth decreased in a dose-dependent manner, and a 2 h exposure to 15% CSE showed sublethal effects (Supplementary Figure S1). After CSE treatment, cells exhibited fragmentation of the vacuole at 0.5 h, and thereafter, it was reduced progressively with time, indicating that vacuolar fragmentation is a transient process that may be affected by the prolonged stress (Figure 1A). To verify that CSE-mediated oxidative stress is the cause of this event, cells were treated with N-acetylcysteine (NAC), a known antioxidant, in conjunction with CSE and then observed for vacuolar morphology. The presence of NAC antagonized the effect of CSE, and the morphology of the vacuole was comparable to wild-type control cells (Figure 1B). This result confirms that CSE-induced oxidative stress is the cause of the CSE-mediated alteration of vacuolar morphology.

### 3.2 Fragmentation of the vacuole aids in combating CSE-induced cytotoxicity

**FIGURE 2:**
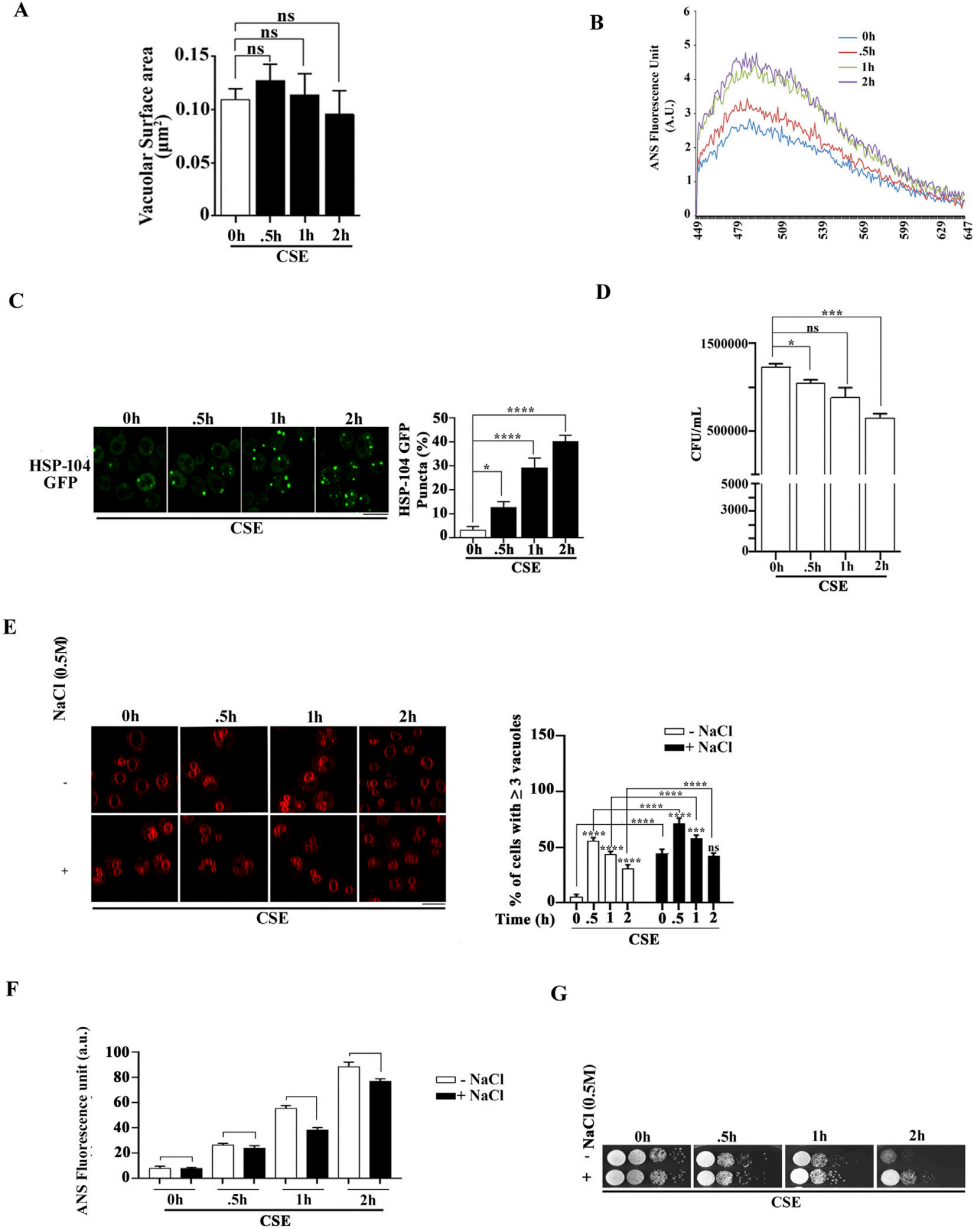
Vacuolar fragmentation helps combat CSE-induced cellular toxicity. **(A)** Vacuolar surface area profile. After CSE treatment, the surface area of vacuoles was calculated using ImageJ software (NIH). For each condition, approximately 10 different fields containing at least 30 cells for each field. **(B)** Misfolded protein profile by ANS staining. Cells were grown until the early exponential phase and adjusted to an OD_600_ of 0.1. The cells were treated with 15% CSE for different time periods and stained with ANS (30 μM) dye, and the level of misfolded protein was spectrofluorimetrically determined. The experiment was performed independently three times, and the given image is representative of the results. **(C)** Misfolded protein profile with HSP-104 GFP. Cells harboring HSP-104-GFP were grown to a midlog phase, treated with CSE for different time periods, and then visualized under a confocal microscope. The bar diagram on the right represents the quantitative profile. **(D)** Cell viability profile. Cells were grown to the early exponential phase and adjusted to an OD_600_ of 0.1 and then treated with 15% CSE for the indicated time periods. In all cases, serial dilutions were spotted on YPD plates, following which the plates were incubated at 30 °C for the indicated time periods (48 or 72 h) before scoring for growth. Each image is representative of 10 different fields. **(E)** NaCl enhances vacuolar fragmentation. Cells were grown till the log phase and were then stained with FM4-64 for 30 min, followed by chasing for 1 h in YPD media. Thereafter, the cells were treated with 0.5 M NaCl for 15 min, followed by CSE treatment for different time periods as indicated and visualized under the microscope. The quantification of the vacuolar number per cell (n ≥ 150 cells per condition). The bar diagram on the right side represents the quantitative profile. **(F)** NaCl reduces misfolded proteins. Cells were grown until the early exponential phase and adjusted to an OD_600_ of 0.1. The cells were treated with 15% CSE for different time periods and stained with ANS (30 μM) dye, and the level of misfolded protein was spectrofluorimetrically determined. **(G)** NaCl enhances cellular viability. Exponentially growing cells treated with CSE in the presence and absence of NaCl for different time intervals were then spotted on YPD agar plates. Each image is representative of ten different fields. Scale bars: 8 μm. Data are presented as the mean ± SD from at least three independent experiments. Statistical analysis was performed using two-way ANOVA, followed by Tukey’s multiple comparisons test. The analysis was done both within conditions and between −NaCl and +NaCl conditions at each time point separately. The horizontal bars show the comparison between the two groups at specific times, whereas the asterisks above bars denote the comparison among different time points in each condition separately. Statistical significance is indicated as follows: n.s., not significant; *p < 0.05; **p < 0.01; ***p < 0.001; ****p < 0.0001. In case of unequal variance **(A–D)**, ANOVA and the Games–Howell test were used. In case of equal variance **(B)**, standard one-way ANOVA and Tukey’s test were used. Significance levels are shown as follows: n. s., not significant; *p < 0.05; **p < 0.01; ***p < 0.001; ****p < 0.0001 (a.u.: arbitrary unit).

The likelihood of fragmentation at the beginning of treatment is to increase the surface area, which may facilitate increased contact between the misfolded protein-harboring endosome and the vacuole, thereby promoting their clearance. To this end, we measured the vacuolar surface area-to-volume ratio after CSE treatment and found that at 0.5 h, the surface area of the vacuole was increased with respect to the control; thereafter, it progressively decreased with time (Figure 2A). To verify whether the increased vacuolar surface area is correlated to better clearance of misfolded protein, we examined the amount of misfolded protein at different time points using ANS dye. The dye monitors misfolded proteins by binding to the exposed hydrophobic regions that become more accessible when proteins unfold or misfold. Thus, this enhanced binding increases the fluorescence intensity and provides a useful tool for measuring misfolded proteins. The results showed that the amount of misfolded protein increased with time and showed an inverse correlation with the vacuolar fragmentation events within the cell (Figure 2B). To further verify the accumulation of misfolded proteins after CSE treatment, yeast cells harboring the HSP104-GFP gene were treated with CSE and thereafter examined for the presence of bright puncta in the cytosol. Hsp104, a disaggregating protein, interacts with misfolded protein aggregates and facilitates either the refolding of these misfolded proteins or their degradation (Parsell et al., 1994). Thus, the presence of cytosolic puncta in the CSE-treated transformant can serve as a marker of aggregated misfolded proteins. Consistent with the ANS data, no or very few green puncta were observed in control cells not treated with CSE. Conversely, CSE-treated cells exhibited more green cytosolic puncta than controls, and the number markedly increased after 1 and 2 h of treatment (Figure 2C). Since increased vacuolar fragmentation upon CSE exposure correlated with reduced misfolded protein levels, we examined cellular survival in relation to vacuolar fragmentation. A cell viability assay was performed, and the results showed the lowest cell death at 0.5 h of CSE treatment; thereafter, cell viability progressively decreased over time (Figure 2D). Thus, these data indicate that vacuole fragmentation is positively correlated with cellular survival.

If vacuolar fragmentation helps combat toxicity, we hypothesize that agents that promote vacuolar fragmentation should exhibit enhanced resistance to CSE-mediated toxicity. To verify this, we pretreated cells with 0.5 M NaCl before exposure to CSE, as NaCl is a known inducer of vacuolar fragmentation (Zieger and Mayer, 2012). In this assay, more vacuolar fragmentation was observed for cells treated with NaCl and CSE than for cells treated only with CSE (Figure 2E). Consistent with this, the cells treated with NaCl and CSE exhibited reduced accumulation of misfolded proteins and better survival (Figures 2F,G) than cells treated only with CSE. All these results bolstered the idea that vacuole fragmentation enhances cellular survival against CSE-induced cellular toxicity.

**FIGURE 3:**
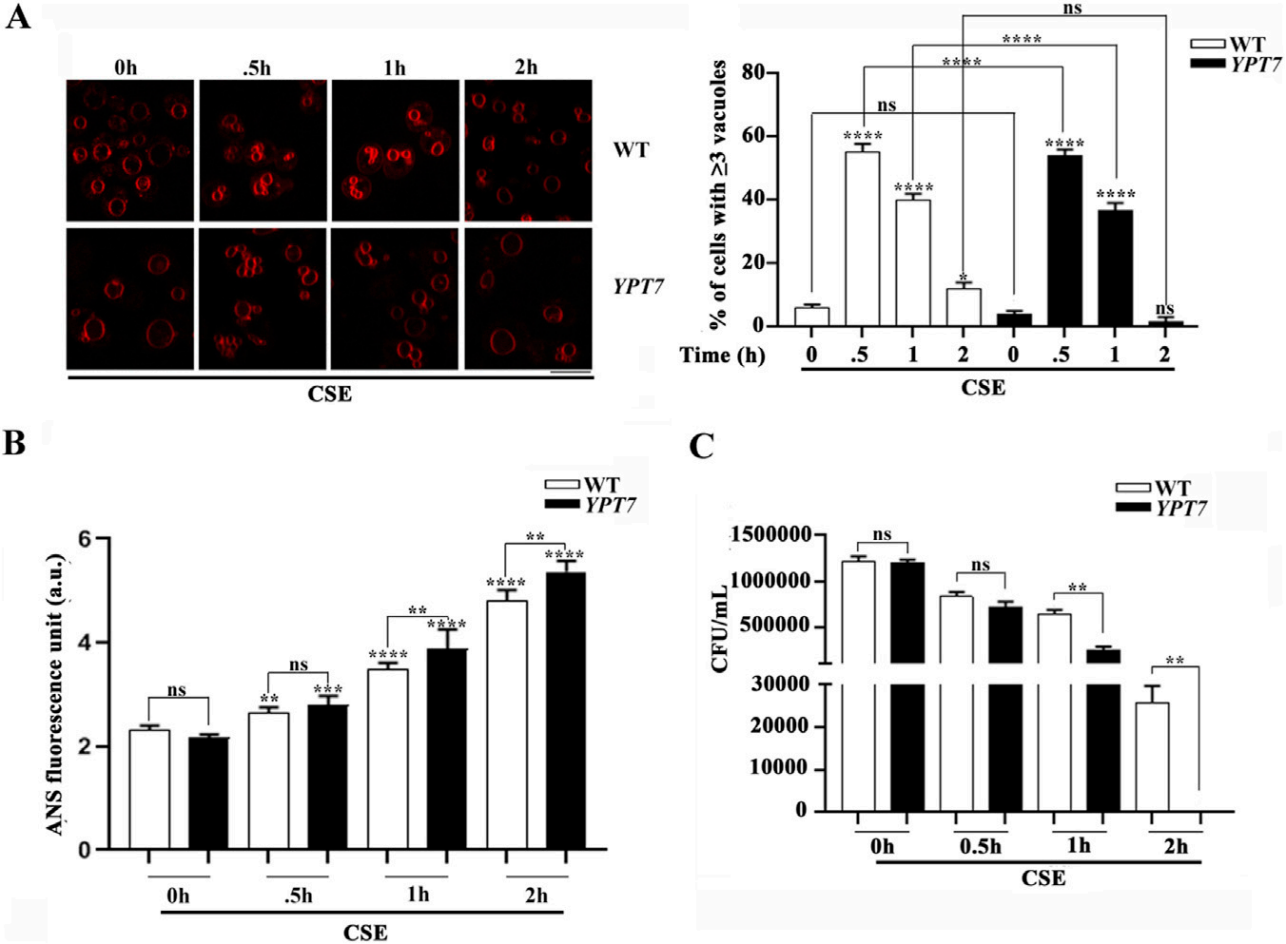
Vacuolar fusion sensitizes cells to CSE. **(A)** CSE-induced vacuolar fragmentation profile of Ypt7-overexpressing cells. Cells harboring or not harboring YPT7 were grown till the log phase and then stained with FM4-64 for 30 min, followed by chasing for 1 h in YPD media, centrifuged, and resuspended in YPD. The cells were then treated with CSE for the indicated time periods and visualized under a microscope. The quantification of the vacuolar number per cell (n ≥ 150 cells per condition). The bar diagram on the right represents the quantitative profile. **(B)** Misfolded protein profile. Cells harboring or not harboring YPT7 were pregrown at 30 °C until the early exponential phase and adjusted to an OD_600_ of 0.1. The cells were then treated with 15% CSE for different time periods as indicated and incubated with 30 μM ANS for 30 min. The level of misfolded protein was spectrofluorimetrically determined. **(C)** CFU profile. Exponentially growing cells harboring or not harboring YPT7 were treated with CSE at different time intervals; the cells were then centrifuged and resuspended in YPD, followed by CSE treatment. Serial dilutions were spotted on YPD plates, after which the plates were incubated at 30 °C for the indicated time period (72 h). Scale bars: 8 μm. Results are expressed as the mean ± SD of three or more independent experiments. The statistical analyses were performed via two-way ANOVA followed by Tukey’s (**A**, **C**) and Sidak (**B**) multiple comparisons test. Comparisons were performed among time points within genotypes and among genotypes within time points. Horizontal lines represent intergroup comparisons, whereas asterisks above bars denote the comparison among different time points in each condition separately. Statistical significance is indicated as follows: n.s., not significant; *p < 0.05; **p < 0.01; ***p < 0.001; ****p < 0.0001 (a.u.: arbitrary unit).

Based on the above observation, we hypothesize that vacuolar fusion will make cells more sensitive to CSE. To verify this, we examined the effects of vacuolar fusion on cell survival upon CSE treatment. For this purpose, yeast cells were transformed with YPT7, which promotes vacuolar fusion, and subsequently treated with CSE for varying periods. Vacuolar morphology, misfolded protein levels, and cell survival were examined as before. At 0.5 h, Ypt7-overexpressing cells exhibited similar levels of vacuolar fragmentation and misfolded protein levels, and consistent with this, they also exhibited similar cellular survival levels compared to wt cells (Figure 3A). However, with the progression of time, vacuolar fragmentation profile changes. At 1 and 2 h, the Ypt7-overexpressing cells exhibited reduced vacuole fragmentation, and consistent with this, higher levels of misfolded protein accumulation and reduced survival were observed compared to wt cells (Figures 3B,C). This result again underscores the importance of vacuolar fragmentation to combat CSE-mediated toxicity.

### 3.3 Excessive fragmentation is detrimental to the cell

**FIGURE 4:**
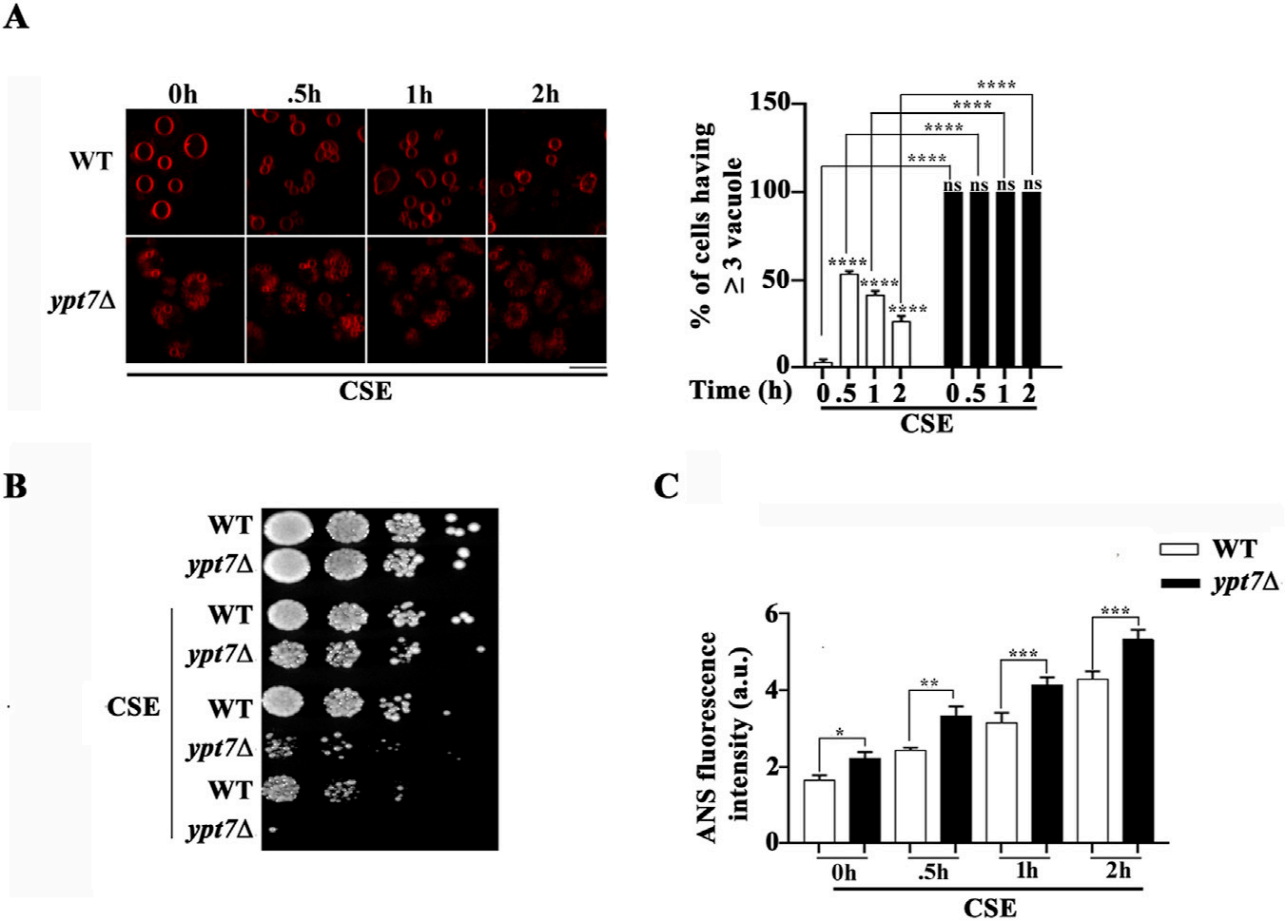
Excessive fragmentation is detrimental for cell survival in CSE-mediated oxidative stress. **(A)** CSE-mediated vacuolar fragmentation profile for ypt7Δ cells. Cells (wt, ypt7Δ) were grown to the log phase, stained with FM4-64 for 30 min, and then chased for 1 h in YPD medium. The cells were then treated with CSE for the indicated time periods and visualized under a microscope. The quantification of the vacuolar number per cell (n ≥ 150 cells per condition). The bar diagram on the right represents the quantitative profile. **(B)** Cell viability profile. Exponentially growing cells (wt, ypt7Δ) were treated with CSE for different time intervals and were then spotted on YPD agar plates. **(C)** Misfolded protein profile. Cells (wt, ypt7Δ) were pregrown at 30 °C until the early exponential phase and adjusted to an OD_600_ of 0.1. The cells were treated with 15% CSE for the indicated time intervals, washed with PBS, and incubated with 30 μM ANS for 30 min. The level of misfolded protein was spectrofluorimetrically determined. Each image represents 10 different fields. Scale bars: 8 μm. Results are expressed as the mean ± SD of three or more independent experiments. The statistical analyses were conducted using two-way ANOVA, followed by Tukey’s multiple comparisons test. Comparisons were made among time points within genotypes and among genotypes within time points. Horizontal lines represent intergroup comparisons, whereas asterisks above bars denote the comparison among different time points in each condition separately. Statistical significance is indicated as follows: n. s., not significant; *p < 0.05; **p < 0.01; ***p < 0.001; ****p < 0.0001 (a.u.: arbitrary unit).

In the previous section, it was shown that vacuolar fragmentation is an important component in combating toxic effects of CSE. In contrast, excessive vacuolar fragmentation has been linked to improper protein sorting across the cell (Raymond et al., 1992). Therefore, we sought to determine the effects of excessive vacuolar fragmentation on cellular susceptibility to CSE treatment. For this purpose, ypt7Δ mutant cells were used, as Ypt7 is known for promoting vacuolar fusion. Consistently, this mutant exhibited excessive fragmentation even without CSE treatment (Figure 4A). Subsequently, vacuolar morphology, cell viability, and misfolded proteins were assessed after CSE treatment for different periods. In all instances, the mutant cells exhibited significantly greater vacuole fragmentation than wt cells. However, despite this high level of vacuolar fragmentation, it was found that mutant cells were more sensitive to CSE than wild-type cells (Figure 4B). Moreover, more misfolded proteins were found in the mutant cells than in wt cells (Figure 4C). These results indicate that there may be a threshold for vacuolar fragmentation above which cell survival is affected by CSE-induced toxicity.

### 3.4 VPS1 and PI(3,5)P2 mediate CSE-induced vacuolar fragmentation

**FIGURE 5:**
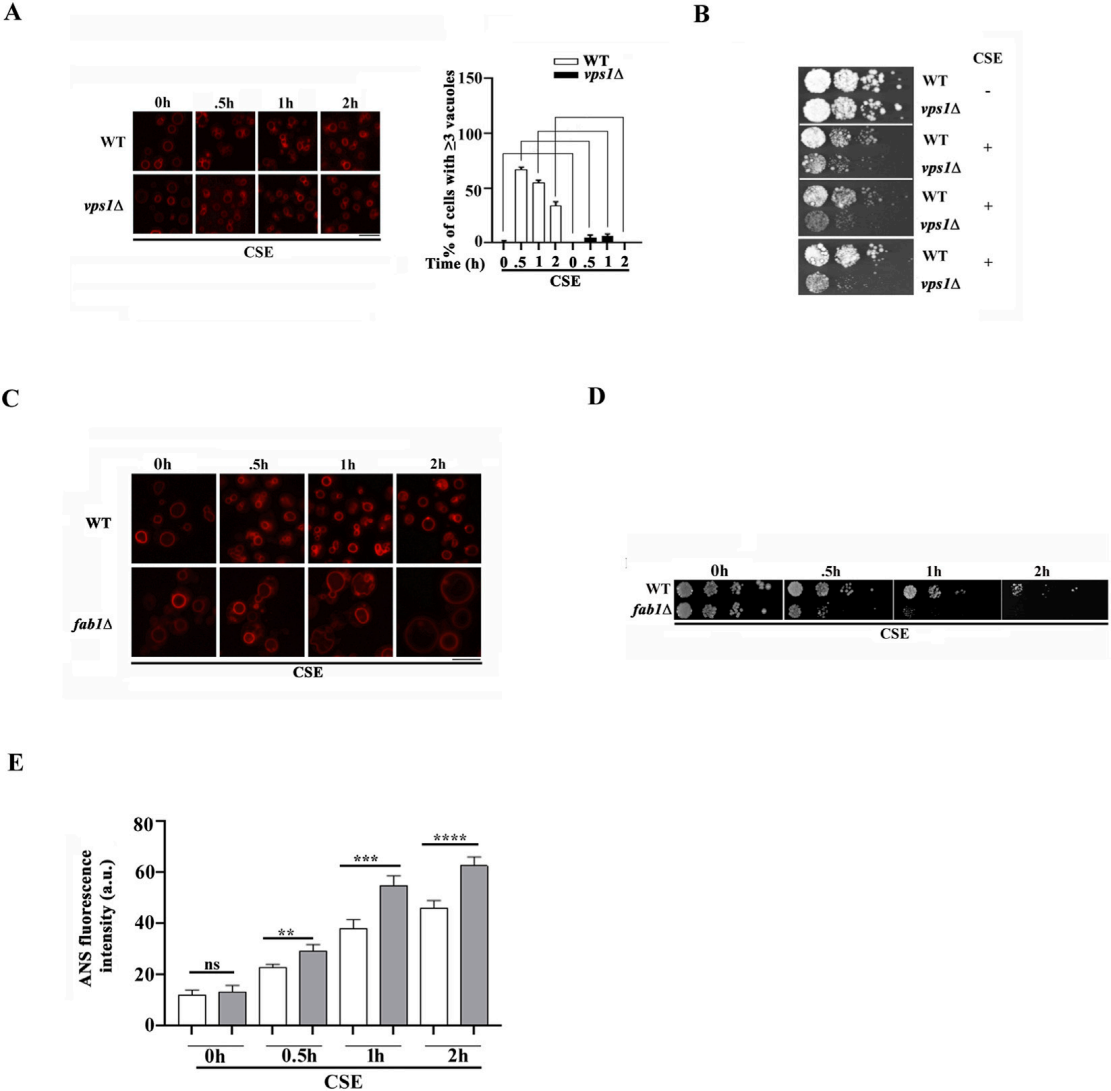
Vps1 and PI(3,5)P2 are involved in CSE-induced vacuole fragmentation. **(A)** Vacuolar fragmentation is Vps1-dependent. Wt and vps1Δcells were grown till the log phase and were then stained with FM4-64 for 30 min, followed by chasing for 1 h in YPD media. Cells were then treated with CSE for different time periods and then visualized under a microscope. The quantification of the vacuolar number per cell (n ≥ 150 cells per condition). The bar diagram on the right side represents the quantitative profile. (**B)** Cell viability profile. Exponentially growing cells (wt, vps1Δ) were treated with CSE for different time intervals and were then spotted on YPD agar plates. Each image represents ten different fields. **(C)** fab1Δ does not undergo vacuolar fragmentation. Cells (wt, fab1Δ) were grown to the midlog phase, stained with FM4-64 for 30 min, and then chased for 1 h in YPD medium. The cells were treated with CSE for different time intervals (0.5, 1, and 2 h) and visualized under a microscope. **(D)** Cell viability assay. Exponentially growing cells (wt, fab1Δ) were treated with CSE for different time intervals and were then spotted on YPD agar plates. **(E)** Misfolded protein profile. Cells were pregrown at 30 °C until the early exponential phase and adjusted to an OD_600_ of 0.1. Cells were treated with 15% CSE for the indicated time intervals, washed with PBS, and incubated with 30 μM ANS for 30 min. The level of misfolded protein was spectrophotometrically determined. Each image represents ten different fields. Scale bars: 8 μm. Data are presented as the mean ± SD from at least three independent experiments. Statistical analysis was performed using two-way ANOVA, followed by Tukey’s multiple comparisons test. Statistical significance is indicated as follows: n. s., not significant; *p < 0.05; **p < 0.01; ***p < 0.001; ****p < 0.0001 (a.u.: arbitrary unit).

During vacuolar fragmentation, Vps1 and PI(3,5)P2 act sequentially and constitute a two-phase process, wherein Vps1 drives initial membrane invagination and PI(3,5)P2 triggers vesicle pinching. Therefore, to gain insight into CSE-induced vacuolar fragmentation, vps1Δcells were treated with CSE, and the vacuolar morphology and CSE sensitivity were examined. The results showed reduced vacuolar fragmentation and reduced cellular survival upon CSE treatment compared to wt cells (Figures 5A,B). These results suggest that CSE-induced vacuolar fragmentation is mediated by Vps1.

To examine the role of PI(3,5)P2, the null mutant of Fab1 (fab1Δ), a kinase that converts PI3P to PI(3,5)P2, was treated with CSE; subsequently, the vacuolar morphology along with their cellular survival was examined. fab1Δ cells exhibited impaired vacuolar fragmentation and showed greater sensitivity to CSE than wt cells (Figures 5C,D). In contrast to vacuolar fragmentation, vacuole size progressively increased over time in fab1Δ mutants. As expected, it was found that misfolded protein load was significantly higher in fab1Δ cells after CSE treatment (Figure 5E).

To evaluate perturbations in vacuolar PtdIns(3,5)P_2_ pools during CSE stress, we employed a sensor of this lipid, SnxA-GFP (Vines et al., 2023). SnxA-GFP localized to the vacuolar membrane at all time points; however, the maximum level was observed at 0.5 h, mirroring the vacuolar fragmentation pattern. This indicates that CSE causes a significant rise in the level of PI(3,5)P2 at 0.5 h to promote vacuolar fragmentation (Supplementary Figure S2). However, prolonged stress reduces the level of PI(3,5)P2 and impairs vacuolar fragmentation. Together, PI(3,5)P2 plays an important role in CSE-mediated vacuolar fragmentation.

### 3.5 Membrane phospholipids are important for vacuolar remodeling

**FIGURE 6:**
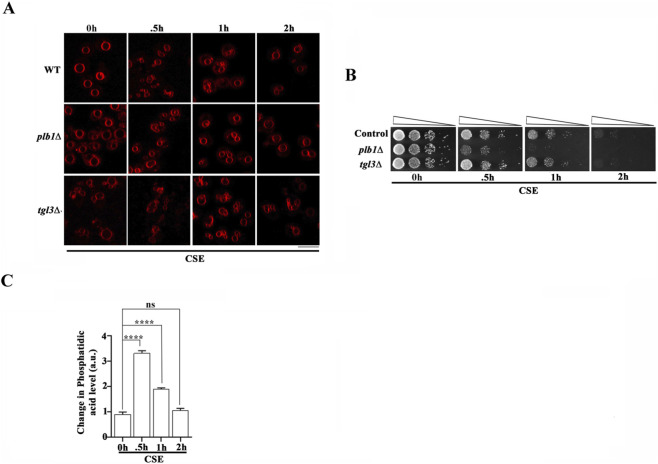
Phospholipase Plb1 plays a crucial role in CSE-induced vacuole fragmentation. **(A)** Vacuolar fragmentation profile for plb1Δand tgl3Δ. Cells (wt, plb1Δ, and tgl3Δ) were grown to the log phase, stained with FM4-64 for 30 min, and then chased for 1 h in YPD medium. The cells were treated with CSE for the indicated time periods and visualized under a microscope. **(B)** Cell viability profile. Exponentially growing cells were treated with CSE for varying durations and then spotted onto YPD agar plates. **(C)** Phosphatidic acid profile. Treated and untreated cell pellets were washed twice with Milli-Q water and were dissolved in methanol: diethyl ether: saturated salt (1:1:1). The diethyl ether fraction was then collected and run on HPLC. Each image represents 10 different fields. Scale bars: 8 μm. Data are presented as the mean ± SD from at least three independent experiments. Statistical analysis was performed using one-way ANOVA, followed by Tukey’s multiple comparisons test. Statistical significance is indicated as follows: n. s., not significant; *p < 0.05; **p < 0.01; ***p < 0.001; ****p < 0.0001.

Vacuole fragmentation requires membrane expansion, which in turn depends on lipid mobilization. The critical sources of lipids for vacuole membrane expansion are triglycerides (storage form) and membrane-bound lipids, which must be mobilized for utilization during vacuole fragmentation. To this end, the current study investigated the cigarette CS-induced vacuole fragmentation in tgl3Δ and plb1Δmutants. Tgl3 is a representative of lipases that hydrolyze stored lipids, and Plb1 is a representative of lipases that hydrolyze phospholipids. The tgl3Δ mutant treated with CSE exhibited a vacuolar fragmentation and growth pattern similar to wt cells (Figures 6A,B). This indicates that the activity of Tgl3 is not required for vacuolar fragmentation. However, it does not rule out the involvement of stored lipids in vacuolar fragmentation, as more isoforms of triglyceride lipase exist to compensate for Tgl3. In contrast, plb1Δ mutants failed to undergo vacuolar fragmentation and exhibited higher sensitivity to CSE than wt cells (Figure 6B). This suggests that phospholipids are critical for generating lipids for vacuolar remodeling.

It is known from the literature that PA is the building block for the synthesis of all the phospholipids. Furthermore, PA is important for membrane curvature and vacuolar fragmentation (Zhukovsky et al., 2019; Burger, 2000). Therefore, PA levels were examined in CSE-treated cells. At 0.5 h, the PA level was significantly increased compared to the no-treatment control (Figure 6C). However, beyond 0.5 h, the PA level progressively decreased, mirroring the temporal pattern of vacuolar fragmentation. Based on this observation, PA may play an important role in CSE-mediated vacuole fragmentation. All these results indicate that membrane phospholipids play a vital role in CSE-induced vacuolar fragmentation.

### 3.6 Vacuolar remodeling requires mitochondrial activity

**FIGURE 7:**
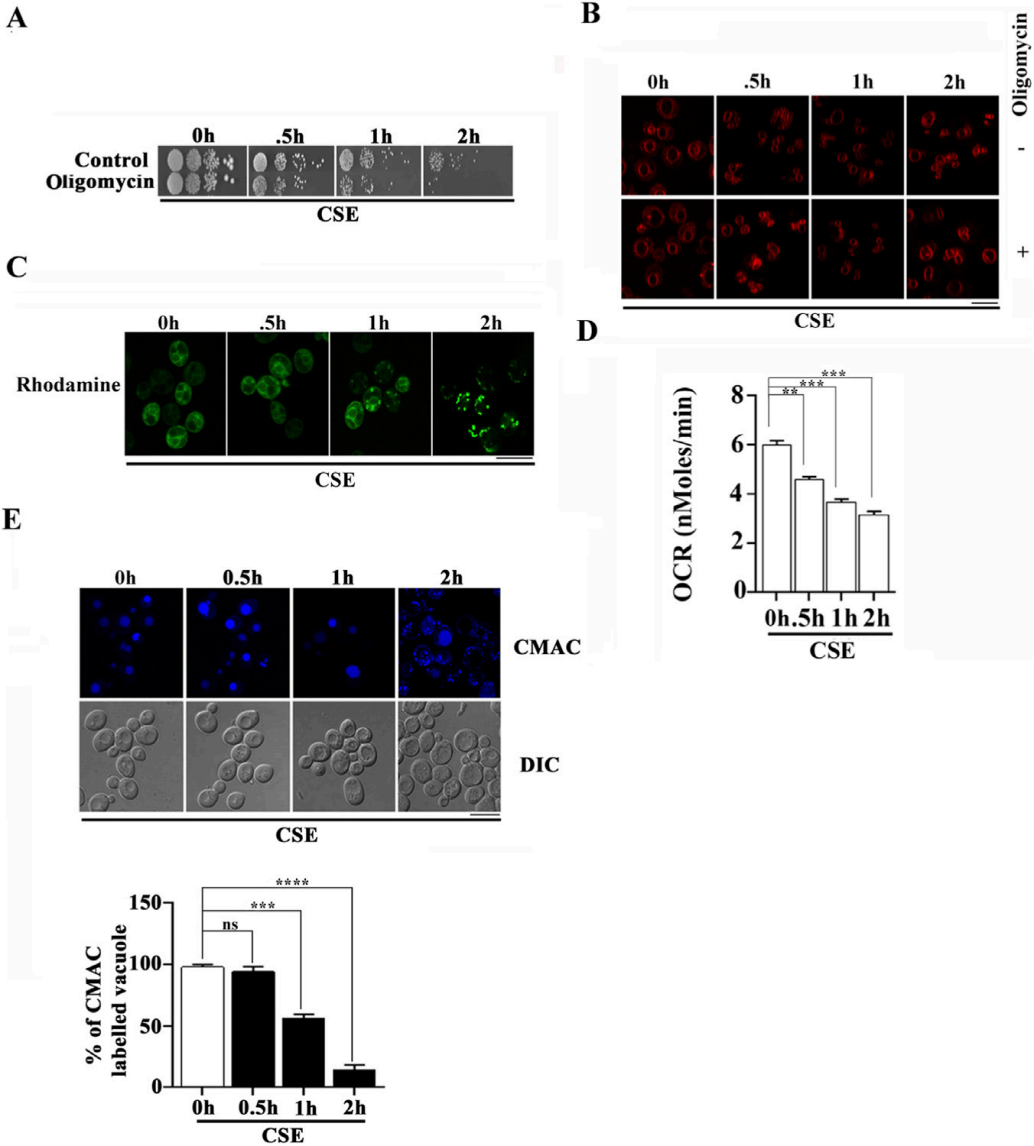
Functional mitochondria are required for CSE-mediated vacuolar fragmentation. **(A)** Cell viability profile. Cells were grown to the midlog phase and treated with 15% CSE for different time periods, as indicated, in the presence and absence of a mitochondrial inhibitor. The cells were then spotted for growth. **(B)** Vacuolar fragmentation profile in the presence of oligomycin. Cells were grown to the midlog phase, stained with FM4-64 for 30 min, and then chased for 1 h in YPD medium. The cells were treated with CSE for different time periods, as indicated, in the presence or absence of oligomycin (10 μM), and visualized under a microscope. (**C)** Rhodamine 123 staining profile. Cells were grown to the midlog phase, treated with CSE for the indicated time periods, stained with Rhodamine 123, and observed under a microscope. **(D)** OCR profile. A cellular OCR was measured for both treated and untreated cells at a concentration of 108 cells/mL using a Clark-type O_2_ electrode. **(E)** CMAC staining profile. Cells were grown to the midlog phase, treated with CSE for the indicated time periods, and then stained with CMAC dye and observed under a microscope. The bar diagram on the right side represents the quantitative profile. Each image represents ten different fields. Scale bars: 8 μm. Data are presented as the mean ± SD from at least three independent experiments. Statistical analysis was performed using one-way ANOVA, followed by Tukey’s multiple comparisons test. Statistical significance is indicated as follows: n.s., not significant; *p < 0.05; **p < 0.01; ***p < 0.001; ****p < 0.0001 (a.u.: arbitrary unit).

Since vacuole fragmentation is an energy-intensive process that requires energy necessary for vesicle transport and membrane remodeling (Baars et al., 2007; Qiu, 2012), the role of mitochondrial respiration in CSE-induced vacuolar fragmentation was investigated. To examine this, cells were treated with the mitochondrial respiration inhibitor oligomycin along with CSE, and their vacuolar morphology as well as sensitivity toward CSE was examined. The oligomycin-treated cells exhibited both excessive sensitivity and reduced vacuolar fragmentation compared to cells treated only with CSE at all time points, with the difference becoming more pronounced after 1 h (Figures 7A,B). The observed difference in fragmentation in the oligomycin-treated cells can be attributed to the reduced availability of energy in these cells, indicating a positive correlation between mitochondrial function and vacuolar fragmentation.

In Figure 1A, reduced vacuolar fragmentation is observed after 1 h of CSE treatment. It raises the possibility that CSE treatment impairs mitochondrial function over time and that the resulting unavailability of ATP leads to decreased fragmentation. To examine this, the mitochondrial membrane potential was assessed by staining CSE-treated and CSE-untreated cells with Rhodamine 123, a dye taken up by mitochondria due to their negative membrane potential, thereby producing green fluorescence. Green fluorescence was observed at all time points, but its intensity and pattern varied. Untreated cells and those treated for 0.5 h displayed filamentous mitochondrial morphology. At 1 h, a mixed phenotype appeared with some cells resembling an untreated control and a substantial population exhibiting punctuated green structures. At 2 h, nearly all cells exhibited punctate mitochondria. The formation of punctate structures, rather than a filamentous pattern, suggests a morphological alteration of mitochondria in CSE-treated cells. It indicates that CSE causes mitochondrial dysfunction, potentially leading to an energy-compromised state (Figure 7C). To gain further support, the OCR was measured before and after CSE treatment of cells and found that the OCR markedly decreased after 0.5 h of CSE treatment (Figure 7D). A lower OCR along with deformed mitochondrial morphology strongly indicates compromised mitochondrial function as mitochondrial function relies on the specific structure of mitochondrial cristae to maximize energy output. When mitochondria become deformed and consume less oxygen, it indicates impairment of the electron transport chain (ETC), resulting in decreased ATP synthesis and compromised cellular energy production.

V-ATPase assembly is required for both vacuolar fragmentation and pH maintenance, and it is energy-dependent. Therefore, a defect in mitochondrial respiration will impair V-ATPase assembly, which in turn perturbs vacuolar pH homeostasis. Vacuolar pH was monitored using CMAC dye, which fluoresces brightly at the physiological pH of the vacuole. CSE-treated cells for 1 h or more showed impaired CMAC staining, indicating altered vacuolar pH. This finding further supports the idea that beyond 0.5 h, CSE treatment impairs mitochondrial function and consequently affects V-ATPase activity, leading to reduced vacuolar fragmentation (Figure 7E).

## 4 Discussion

This study demonstrates that CSE induces rapid, transient vacuolar fragmentation in *S. cerevisiae* and that this response serves as an adaptive cytoprotective mechanism against smoke-induced cellular stress. Our findings reveal that vacuolar remodeling is tightly coordinated with proteostasis, phospholipid metabolism, and mitochondrial activity, thereby highlighting the central role of the vacuole in cellular adaptation to oxidative stress.

CS is a complex mixture containing many oxidants and reactive chemicals capable of disrupting cellular homeostasis. Consistent with this, the treatment of yeast cells with CSE resulted in rapid vacuolar fragmentation within 0.5 h of exposure. The observation that antioxidant treatment with N-acetylcysteine abolished this phenotype strongly indicates that oxidative stress is the primary trigger for vacuolar remodeling. Previous studies have shown that environmental stresses such as hyperosmotic stress, nutrient limitation, and oxidative insults induce vacuolar fission as part of a conserved stress adaptation program (Stauffer and Powers, 2015). Our findings extend these observations by identifying CS-derived oxidative stress as another potent inducer of vacuolar fragmentation.

An important finding of this study is the transient nature of the fragmentation response. Vacuolar fragmentation peaked at 0.5 h and subsequently declined despite continued exposure to CSE. This suggests that vacuolar fission represents an early adaptive response rather than a permanent morphological state. Quantitative analysis demonstrated that increased vacuolar fragmentation was associated with a higher vacuolar surface area-to-volume ratio. Such remodeling likely enhances membrane contact opportunities between the vacuole and endosomal compartments, thereby facilitating trafficking and degradation of damaged cellular constituents (Hirata et al., 2023). The inverse correlation observed between vacuolar fragmentation and misfolded protein accumulation supports this interpretation. Cells displaying higher fragmentation contained lower levels of ANS-positive misfolded proteins and fewer Hsp104-positive aggregates, indicating more efficient clearance of proteotoxic species.

The functional significance of vacuolar fragmentation was further demonstrated by survival analyses. Cell viability was highest at the time point exhibiting maximum fragmentation and progressively declined as fragmentation diminished. Furthermore, the artificial enhancement of fragmentation through NaCl pretreatment reduced misfolded protein accumulation and improved survival during CSE exposure. Conversely, the promotion of vacuolar fusion via YPT7 overexpression increased the proteotoxic burden and reduced viability. Together, these findings support a model in which controlled vacuolar fragmentation enhances cellular resilience by promoting the removal of damaged proteins generated during oxidative stress.

Although vacuolar fragmentation is beneficial, our results indicate that excessive fragmentation becomes detrimental. The ypt7Δ mutant, characterized by constitutively fragmented vacuoles, displayed increased sensitivity to CSE despite exhibiting greater fragmentation than wild-type cells. These cells accumulated more misfolded proteins and showed reduced survival. This observation suggests that vacuolar morphology must be maintained within an optimal range and that both insufficient and excessive fragmentation impair cellular fitness. It has been previously shown that excessive fragmentation was associated with defects in vacuolar inheritance, trafficking, and protein sorting (REF). Therefore, while moderate fragmentation may increase degradative capacity, excessive fragmentation may compromise vacuolar functionality and offset any protective benefit. These findings suggest a threshold beyond which fragmentation becomes maladaptive.

The molecular basis of CSE-induced vacuolar remodeling appears to involve the canonical vacuolar fission machinery. Both Vps1 and Fab1 were required for efficient fragmentation following CSE treatment. Vps1, a dynamin-like GTPase, mediates membrane constriction during vacuolar fission, whereas Fab1 generates phosphatidylinositol-3,5-bisphosphate [PI(3,5)P_2_], a signaling lipid essential for membrane scission (Weisman, 2006; Dove et al., 1997; Malia et al., 2018; Chen et al., 2021). Mutants lacking either VPS1 or FAB1 exhibited impaired fragmentation, increased accumulation of misfolded proteins, and heightened sensitivity to CSE. These results establish that CS-induced vacuolar remodeling uses conserved vacuolar fission pathways.

Further evidence for the involvement of PI(3,5)P2 was provided by the SnxA-GFP biosensor. Accumulation of PI(3,5)P2 on vacuolar membranes was maximal at 0.5 h, closely paralleling the kinetics of vacuolar fragmentation. Since PI(3,5)P_2_ is known to regulate membrane dynamics and vacuolar morphology under stress conditions, the transient increase observed here likely represents a key signaling event initiating fragmentation (Weisman, 2006; Dove et al., 1997; Malia et al., 2018; Chen et al., 2021). The subsequent decline in PI(3,5)P2 levels may contribute to the reduction in fragmentation observed during prolonged CSE exposure.

Membrane lipid remodeling emerged as another critical determinant of vacuolar fragmentation. Vacuolar expansion and fission require substantial membrane reorganization and therefore depend on the availability of lipid precursors. Interestingly, deletion of TGL3, a major triglyceride lipase, did not significantly affect fragmentation, suggesting that mobilization of storage lipids is not the primary source of membrane material during CSE stress. In contrast, deletion of PLB1 markedly impaired fragmentation and increased sensitivity to CSE, indicating a crucial role for phospholipid turnover. These findings suggest that membrane phospholipids serve as the principal lipid reservoir supporting vacuolar remodeling during oxidative stress. This was further supported by the observation that PA levels significantly increased during the early phase of CSE exposure and subsequently declined in parallel with vacuolar fragmentation. PA occupies a central position in phospholipid metabolism and possesses intrinsic biophysical properties that promote membrane curvature and fission. Previous studies have implicated PA in vacuolar membrane dynamics and organelle remodeling (Burger, 2000; Zhukovsky et al., 2019). Therefore, the transient elevation of PA observed in this study may facilitate membrane deformation and support the generation of fragmented vacuolar structures. Collectively, these results identify phospholipid metabolism and PA production as important contributors to the vacuolar adaptive response.

Another major finding of this study is the dependence of vacuolar remodeling on mitochondrial function. Inhibition of oxidative phosphorylation by oligomycin significantly reduced vacuolar fragmentation and increased cellular sensitivity to CSE, indicating that vacuolar fission is an energy-dependent process (Baars et al., 2007; Qiu, 2012). Vacuolar remodeling requires ATP-driven membrane trafficking, lipid mobilization, and the operation of vacuolar proton pumps; therefore, mitochondrial ATP production is likely essential to sustain these processes.

The temporal decline in vacuolar fragmentation prompted us to investigate whether prolonged CSE exposure compromises mitochondrial activity. Several observations support this possibility. First, mitochondrial morphology progressively shifted from a filamentous network to fragmented punctate structures, a hallmark of mitochondrial dysfunction. Second, the OCR decreased following CSE treatment, indicating impaired respiratory activity. Together, these findings suggest that CS progressively damages mitochondrial function, thereby limiting cellular energy availability. As mitochondrial dysfunction develops, the energy-demanding process of vacuolar fragmentation may no longer be sustained, resulting in reduced vacuolar remodeling and diminished clearance of damaged proteins.

Mitochondrial dysfunction may further influence vacuolar physiology by disrupting V-ATPase assembly and activity. Proper V-ATPase function requires adequate ATP supply and is essential for vacuolar acidification, protein degradation, and membrane dynamics. Consistent with this notion, prolonged CSE exposure impaired CMAC staining, indicating disruption of vacuolar pH homeostasis. Since vacuolar acidification is required for both degradative function and vacuolar membrane remodeling, mitochondrial impairment likely contributes indirectly to reduced fragmentation by compromising V-ATPase-dependent processes. Thus, mitochondrial dysfunction may represent a critical turning point at which an initially adaptive response transitions into cellular failure.

Together, our findings support a model in which CSE-induced oxidative stress rapidly activates Vps1- and PI(3,5)P_2_-dependent vacuolar fragmentation. This early remodeling event increases the vacuolar membrane surface area, facilitates the clearance of misfolded proteins, and promotes cellular survival. The process depends on phospholipid-derived membrane remodeling and adequate mitochondrial energy production. However, prolonged CSE exposure progressively impairs mitochondrial function, reduces ATP availability, disrupts vacuolar acidification, and diminishes vacuolar fragmentation. Consequently, damaged proteins accumulate, proteostasis collapses, and cellular viability declines. Importantly, both insufficient and excessive fragmentation are detrimental, indicating that the optimal regulation of vacuolar morphology is essential for stress adaptation.

## 5 Conclusion

This study identifies vacuolar fragmentation as a previously underappreciated protective mechanism against CS-induced cellular toxicity. The findings show an intricate interplay among oxidative stress signaling, vacuolar dynamics, phospholipid metabolism, and mitochondrial function in determining cellular fate during smoke exposure. These observations not only advance our understanding of vacuolar stress responses in yeast but may also provide broader insights into lysosome-mediated adaptive mechanisms relevant to oxidative stress-associated diseases in higher eukaryotes.

## Data Availability

The original contributions presented in the study are included in the article/Supplementary Material; further inquiries can be directed to the corresponding author.
