# Supplementary material for: Treatment of Saccharomyces cerevisiae with cigarette smoke extract causes vacuolar fragmentation to combat cigarette smoke-induced cellular toxicity
Source: Front Toxicol. 2026 Jul 22;8:1835170. doi: 10.3389/ftox.2026.1835170 (PMC13439676; doi:10.3389/ftox.2026.1835170)
Supplement: Supplementary file 1 [file DataSheet1.docx]

**Supplementary materials**

**Supplementary Figure S1**. **CSE dose-response kinetics.** Exponentially growing *S. cerevisiae* cells were treated or not treated with different doses of CSE for different time periods, as shown in the figure. Cells were then spotted on YPD agar plates and incubated for 48 h.

**Supplementary Figure S2. Evaluation of vacuolar pool of PI3,5 P2 levels during CSE treatment.** Cells expressing SnxA-GFP were grown to mid-log Phase and treated with 15% CSE for the indicated time periods, as shown in the figure. Cells were then washed and visualized under the microscope. SnxA-GFP, which is a sensor for PI3,5 P2 (Vines et al. 2023), was found on the vacuolar membrane at 0 and 0.5 h; however, no or very little SnxA-GFP was present on the vacuolar membrane at 2 h. The intensity of SnxA-GFP peaked at 0.5 h and then gradually decreased. Scale bar: 6 µm.
